# Rare Earth Element Extraction from Ionic Rare Earth Ores by Two Typical Acidogenic Microorganisms, *Aspergillus niger* and *Acidithiobacillus ferrooxidans*

**DOI:** 10.3390/ijms26051986

**Published:** 2025-02-25

**Authors:** Mengyuan Wang, Jingna Li, Hongchang Liu, Shiyun Huang, Xiaoyan Liu, Yang Liu, Muhammad Awais, Jun Wang

**Affiliations:** 1 School of Minerals Processing and Bioengineering, Central South University, Changsha 410083, China; mengyuanwang@csu.edu.cn (M.W.); li_jingna@csu.edu.cn (J.L.); hsy2023@csu.edu.cn (S.H.); liuxiaoyan20010228@163.com (X.L.); liuyang_feiyang@163.com (Y.L.); muhammadawaissagar786@gmail.com (M.A.); wjwq2000@126.com (J.W.); 2 Key Lab of Biometallurgy of Ministry of Education of China, Central South University, Changsha 410083, China

**Keywords:** ionic rare earth ore, microbe-mineral interaction, *Aspergillus niger*, *Acidithiobacillus ferrooxidans*, comparative transcriptomics

## Abstract

Ionic rare earth ore (IREO) has a high abundance of medium and heavy rare earth elements (REEs), making it a vital strategic resource for China. In this work, two typical microorganisms, *Aspergillus niger* and *Acidithiobacillus ferrooxidans*, were used to study the interaction mechanism during the bioleaching of IREO under acidic conditions. The results revealed some differences in the interaction and leaching effects of *A. niger* and *A. ferrooxidans* on ionic rare earth minerals. *A. niger* mainly forms rare earth complexes with rare earth ions in IREO by secreting metabolites such as organic acids, thereby promoting the release of REEs, and it has a strong adsorption capacity for Yb. *A. ferrooxidans* promotes the release of REEs from rare earth minerals, primarily through iron–sulfur oxidation. The differential expression of metabolic genes (e.g., *gpmL*, *FabF*, *FASN*) associated with major metabolite secretion indicates their correlation with the leaching process. The above results reveal the role of the typical acid-producing microorganisms *A. niger* and *A. ferrooxidans* and their metabolites in the leaching of IREO, which is valuable for understanding the interaction mechanisms between microorganisms and IREO under acidic conditions.

## 1. Introduction

Rare earth elements (REEs) include the lanthanides found in the periodic table, specifically those with atomic numbers ranging from 57 to 71, as well as scandium (Sc, atomic number 21) and yttrium (Y, atomic number 39). REEs can be divided into light REEs and heavy REEs according to differences in their atomic number and atomic mass [1,2]. REEs are widely used in metallurgy, the petrochemical industry, special glass, precision ceramics, catalysis, high-temperature superconductors, new photoelectric magnetic materials, and other fields, and they have a reputation as “industrial vitamins” [3,4,5]. REEs exist naturally in a variety of minerals, such as cerium fluoride, monazite, phosphate rock, ionic rare earth ore (IREO), coal fly ash, and solution waste [6,7,8,9,10,11,12]. Among these, IREO is an important source of medium and heavy REEs. Many traditional methods have been studied for the extraction of IREO, such as ammonium or magnesium sulfate solutions as leaching solutions for REEs via in situ leaching, ammonium chloride precipitation [13,14], the solvent extraction method [15], and the impregnated resin method for the enrichment of REEs [16]. These methods are complex and can easily cause environmental pollution. To address these issues, bioleaching has been proposed for REE extraction. The bioleaching technique is widely recognized in the mining sector, particularly for the recovery of elements from metal sulfide ores. Using this method to extract metals can result in greater ecological benefits [17]. This promising technology for extracting REEs from IREO has been studied by an increasing number of researchers.

The bioleaching method is based on three principles—acid hydrolysis, complexation, and redox—used to effectively leach REEs. Microorganisms and their metabolites are crucial in the leaching process of REEs. For example, heterotrophic microorganisms form organic acids, iron carriers, extracellular polymers, and other metabolites through the metabolic transformation of sugar compounds to dissolve metal ores [18,19,20]. The primary metabolites generated by microorganisms are organic acids. By complexing with metals, soluble organometallic complexes can be formed by reducing the pH of the solution [17]. Bioleaching has unique advantages over chemical leaching. Chemical leaching is achieved mainly through the use of high concentrations of acids or salts and produces waste solvents that highly pollute the environment. In addition, a wide range of REEs are mixed in the ore; therefore, each chemical separation results in an overall inefficiency of recovery [21,22]. Bioleaching can be a solution to these problems in chemical leaching processes. Bioleaching is environmentally friendly and more selective in the extraction of REEs from IREO [23]. For example, *Bacillus subtilis* can selectively accumulate REEs from solutions containing Fe(II) [24], and *Myxococcus xanthus* can selectively adsorb La via polysaccharides in extracellular polymers [25].

One restriction in the application of REE bioleaching is its relatively slow leaching efficiency compared with that of chemical leaching. Therefore, most current studies have focused on the leaching rates of REEs and the differences in the leaching of rare earth minerals by different microorganisms/metabolites. For example, Shen et al. comparatively studied the noncontact bioleaching of *Aspergillus niger* and *Yarrowia lipolytica* with IREOs, and the results revealed that *A. niger* metabolites were more effective at leaching rare earth ions [26]. In the experiments of Megan et al., the leaching rates of IREOs using the heterotrophic microbial isolates *Aspergillus* and *Bacillus* were compared, and the release rate of rare earth ions was inversely proportional to the pH value (for Lu), which suggested that acidic conditions may be more favorable for the bioleaching of rare earth ions [27]. In addition, some studies propose the extraction of REEs via microbial fermentation metabolites. For example, citric acid, malic acid, and other organic acid metabolites produced via *A. niger* metabolism can be used to extract REEs through acidolysis, complexation, etc. [26]. *Acidithiobacillus ferrooxidans* is an iron- and sulfur-oxidizing bacterium that can oxidize Fe(II) into Fe(III) and reduce sulfur species to sulfuric acid; thus, it is also an excellent leaching agent that can effectively leach REEs from waste fluid catalytic cracking catalysts [28]. These studies revealed that *A. niger* extracted rare earth ions effectively through acidolysis and complexation because the organic acids contained in the metabolites improved their leaching. *A. ferrooxidans* is also effective in extracting rare earth ions from its structure. Although these studies are valuable for understanding the bioleaching of REEs, the underlying mechanisms of the microbial and metabolite interactions with IREO remain unclear in theory. Understanding these mechanisms is crucial for improving rare earth leaching processes and promoting the development of microbial extraction technology for REEs from IREO.

To study the relevant mechanism of the interaction between acidogenic microorganisms and IREO, *A. niger* and *A. ferrooxidans* were selected as representative microorganisms to establish an interaction system with IREO. The influences of microbial cells and microbial metabolites on the dissolution of REEs were comparatively analyzed. The morphology, structure, distribution of REEs, functional groups, and metabolic pathways of the cell and mineral phases were analyzed by scanning electron microscopy (SEM), transmission electron microscopy (TEM), X-ray diffraction (XRD), Fourier transform infrared spectroscopy (FTIR), comparative transcriptomics, and metabonomics. The results revealed the similarities and differences in the interactions between *A. niger* or *A. ferrooxidans* and IREO and are valuable for elucidating the leaching mechanisms of these two microorganisms and their metabolites when interacting with ionic rare earth minerals, laying the foundation for future applications of environmentally friendly IREO bioleaching technology in the industry.

## 2. Results

### 2.1. Solution Behavior

#### 2.1.1. Cell Concentration, pH, and ORP Changes

**Figure 1:**
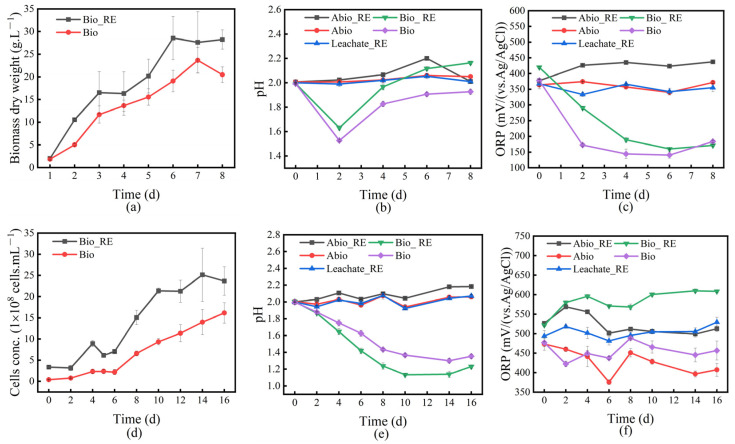
Changes in the cell concentration, solution pH, and ORP during the interaction of *A. niger* (**a**–**c**) and *A. ferrooxidans* (**d**–**f**) with ionic rare earth minerals.

The changes in the solution behavior of the Bio_RE group and the Bio group were analyzed during the interaction of *A. niger* and *A. ferrooxidans* with IREO, and the results are shown in Figure 1. Figure 1a displays the biomass change curves of *A. niger* with and without rare earth minerals. As shown in Figure 1a,d, the cell concentration in the presence of rare earth minerals during culture was significantly greater than that in the Bio group without rare earth minerals. The results indicate that rare earth minerals can promote the growth of *A. ferrooxidans* and *A. niger*.

**Figure 2:**
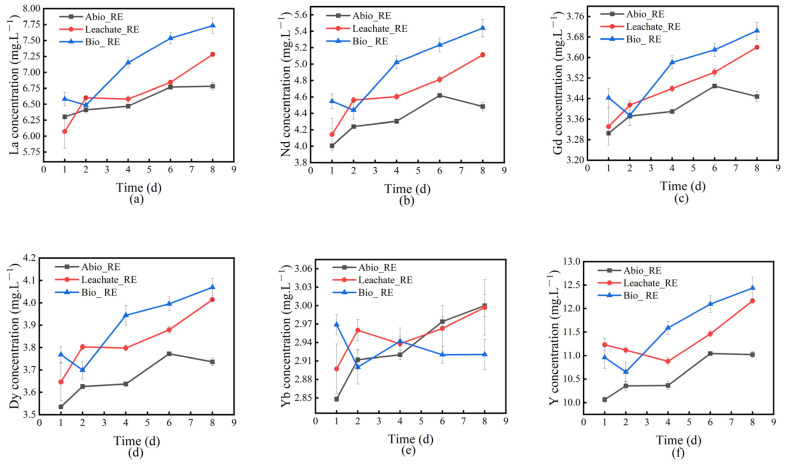
Changes in the concentration curves of La (**a**), Nd (**b**), Gd (**c**), Dy (**d**), Yb (**e**), and Y (**f**) in the solution of the *A. niger*—rare earth ore interaction system.

Figure 1b illustrates that during the interaction between *A. niger* and IREO, the pH of the solution decreased to the lowest value on day 2, when the organic acid content produced by *A. niger* was the highest, and then, the pH value increased, indicating that the growth period of *A. niger* was terminated. The increase in pH in the medium may be the result of cell wall lysis, the release of intracellular metabolites, and alkaline buffering. As shown in Figure 1b, the pH value of the Bio_RE group was always greater than that of the Bio group, indicating that the presence of rare earth minerals might inhibit the release of *A. niger* acid metabolites to a certain extent. Figure 1e shows the pH change in the solution during the interaction between *A. ferrooxidans* and IREO, revealing that the pH decreased to the lowest value on day 10 and then reached a stable state, and the changing trend may be related to the growth state of *A. ferrooxidans*. In the presence of rare earth minerals, the pH value was lower. The growth curve of *A. ferrooxidans* shown in Figure 2d indicates that the presence of rare earth minerals can promote the growth of *A. ferrooxidans* and the release of acidic substances. Figure 2c shows the change in the redox potential during the interaction between *A. niger* and ionic rare earth minerals. With increasing leaching time, the redox potential gradually decreased; that is, the reducibility gradually increased. The presence of rare earth minerals weakened the reducibility of the solution system to a certain extent.

#### 2.1.2. Changes in Rare Earth Ion Concentrations

**Figure 3:**
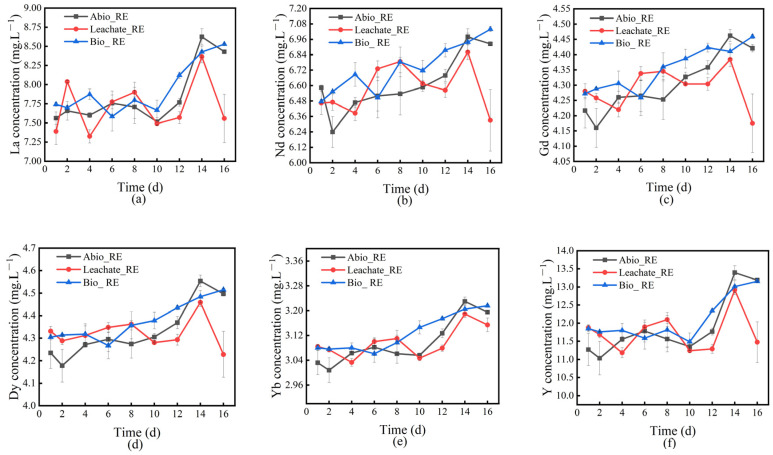
Changes in the concentration curves of La (**a**), Nd (**b**), Gd (**c**), Dy (**d**), Yb (**e**), and Y (**f**) in the solution of the *A. ferrooxidans*—rare earth ore interaction system.

We measured the concentrations of rare earth ions in different groups of solution systems during the interaction between *A. niger* and *A. ferrooxidans* and IREOs, and the results are shown in Figure 2 and Figure 3. As shown in Figure 2, *A. niger* and its fermentation dissolved most of the rare earth ions in IREO, and the concentration of Al in the solution continued to increase with increasing time (see details in Figure S1), whereas the concentrations of REEs, except for Tb and Tm, in the solution decreased on day 2, possibly due to the early stage of the *A. niger* culture. The biomass and organic acid metabolites of *A. niger* were relatively low, the adsorption and dissolution effects of rare earth ions were weak, and the clay minerals themselves adsorbed rare earth ions [29]. After the rare earth ions in the rare earth minerals were dissolved by the organic acids and other metabolites secreted by *A. niger* on day 1, the dissolution effect of the REEs weakened because of their low content, and the rare earth ions in the solution were reversely adsorbed to the clay minerals. The contents of Tb and Tm did not decrease significantly, possibly owing to the weak adsorption of the clay minerals. From days 4 to 8 of leaching, the concentrations of Sm, Eu, Tb, Ho, Er, Tm, and Yb of several REEs first decreased and then increased, possibly because of the adsorption of REEs by the mycelium and IREO of *A. niger* in the late stage of the experiment, corresponding to a decrease in the REE concentration in the solution. Extracellular substances, such as the organic acids produced by *A. niger*, can promote the release of REEs in minerals, increasing the concentration of REEs in the solution. Moreover, the concentration of REEs in the Bio_RE solution fluctuated more than that in the Leachate_RE group did; that is, the concentration of REEs in the solution decreased more and increased faster, indicating that *A. niger*, the organic acids, and other metabolites produced by *A. niger* had competitive adsorption effects on the REEs in the solution, and the organic acids and other metabolites produced by *A. niger* can significantly promote the release of the REEs in ionic rare earth minerals [30]. In particular, the concentration of Yb in the Bio_RE group solution did not increase after it began to decrease at day 4, whereas the concentration of Yb in the Leachate_RE group solution increased after a short decline, indicating that *A. niger* had a strong adsorption capacity for Yb.

Figure 3 shows the change in the REE concentration in the solution during the interaction between *A. ferrooxidans* and IREO. The results indicated that the concentration of REEs in the solution did not increase significantly during the first 6 d of leaching. After 6 d, with increasing leaching time, the concentration of *A. ferrooxidans* oxide increased rapidly. The concentration of REEs in the solution of the Bio_RE group increased rapidly and was significantly greater than that of the Leachate_RE group. The results revealed that the dissolution of REEs was positively correlated with the concentration of *A. ferrooxidans*. This is mainly because *A. ferrooxidans* can effectively oxidize ferrous ions (Fe^2+^) to iron ions (Fe^3+^) in ores through their strong oxidizing ability, and the resulting Fe^3+^ can form complexes with REEs, thereby promoting their dissolution. In addition, the sulfuric acid produced by *A. ferrooxidans* during metabolism lowers the pH of the solution, creating an acidic environment conducive to the desorption and dissolution of REEs, which, in turn, increases their solubility. Moreover, relatively high concentrations of *A. ferrooxidans* can increase the release of metabolites, such as extracellular polymers, which not only increase the availability of REEs through bioabsorption but also optimize the nutritional conditions of the ore, promoting microbial growth and competitive advantages [31]. At the later stage of leaching, the concentrations of Pr, Sm, Eu, Tb, Ho, Tm, and Lu in the solution decreased significantly, whereas the concentrations of La, Ce, Nd, Gd, Dy, Er, Yb, and Y continued to increase. As shown in Figure 3, the concentration of REEs in the solution of the interaction system between *A. ferrooxidans* and ionic rare earth minerals changed. The change trends of the other elements were the same, indicating that the adsorption effect of ionic rare earth minerals on Pr, Sm, Eu, Tb, Ho, Tm, and Lu was strong. During the interaction between *A. niger* and *A. ferrooxidans* and ionic rare earth minerals, the concentration of rare earth ions was relatively high in the microbial system (Bio_RE group), indicating that the presence of microorganisms is conducive to the bioleaching of REEs [32], possibly because the microorganisms themselves and their metabolites play important roles in the leaching of REEs. Moreover, the presence of microorganisms can enhance leaching by mitigating the precipitation pathway of dissolved REEs [17].

**Figure 4:**
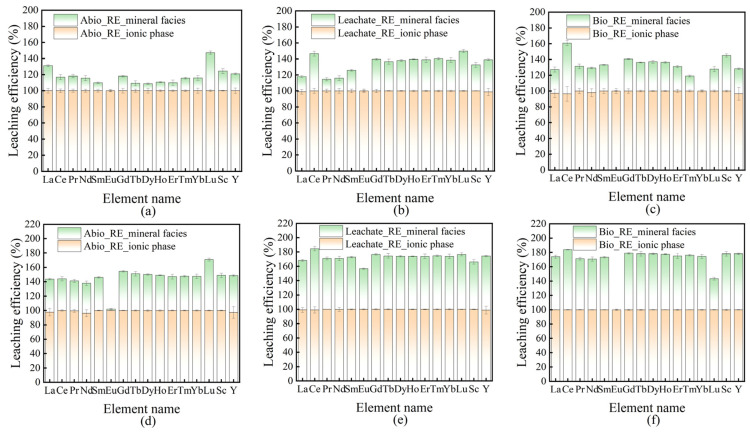
The leaching rates of rare earth ions in the solution compared to those of original rare earth minerals during the interaction between *A. niger* (**a**–**c**)/*A. ferrooxidans* (**d**–**f**) and rare earth minerals.

The leaching rates of REEs in the solution of the interaction system between *A. niger* and *A. ferrooxidans* and ionic rare earth minerals were calculated, and the results are shown in Figure 4. The results indicate that the REEs dissolved in the interaction between *A. niger* and *A. ferrooxidans* and ionic rare earth minerals can promote the release of mineral and colloidal REEs in addition to the ionic phase and water-soluble phase.

Among the Abio_RE, Leachate_RE, and Bio_RE groups of *A. niger*, the leaching rates of La in the Leachate_RE group were lower, and the leaching rates of Ce, Sm, Gd, Tb, Dy, Ho, Er, Tm, Yb, Sc, and Y were higher. The overall leaching rate increased, indicating that the metabolites of *A. niger* played an important role in the leaching of rare earth ions in the mineral phase. Compared to those in the Abio_RE group, the leaching rates of La in the Bio_RE group also decreased, whereas the leaching rates of Ce, Pr, Nd, Sm, Gd, Tb, Dy, Ho, Er, Tm, Sc, and Y increased, indicating that the presence of *A. niger* and its metabolites had a positive effect on the leaching of REEs other than La. Between the Leachate_RE and Bio_RE groups of *A. niger*, the leaching rates of four elements (La, Ce, Pr, and Nd) in the Bio_RE group of *A. niger* increased, whereas the leaching rates of five elements (Er, Tm, Yb, Lu, and Y) decreased, indicating that *A. niger* metabolites play an important role in the leaching of heavy rare earth ions. This may be due to the action of organic acids in the metabolites of *A. niger*, which have stronger complexation effects on heavy REEs than on light REEs, and because of the acidity of organic acids, it is easier to release heavy REEs locked in the mineral matrix [27]. *A. niger* itself plays an important role in the leaching of light and medium REEs, but it is not conducive to the bioleaching of heavy REEs, which may be due to the strong absorption of heavy rare earth ions by *A. niger* filaments, which is not conducive to the release of heavy rare earth ions into the solution.

Among the Abio_RE, Leachate_RE, and Bio_RE groups of *A. ferrooxidans,* the Leachate_RE group presented a greater leaching rate of all REEs than the Abio_RE group. The leaching rates of all the REEs, except Lu and Eu, increased in the Bio_RE group of *A. ferrooxidans*. The leaching rates of all REEs, except Lu and Eu, in the Bio_RE group were greater than those in the Leachate_RE group. In the presence of *A. ferrooxidans*, the overall leaching rates of REEs were relatively high but not favorable for the leaching of Eu and Lu.

During the interaction between *A. niger*/*A. ferrooxidans* and IREO, the maximum leaching rate of *A. ferrooxidans* was 94.03%, and that of *A. niger* was 83.48% (the specific results are shown in Figure S2), suggesting that the dissolution rate of the REEs in the solution of *A. ferrooxidans* and the IREO interaction system was greater than that of *A. niger*.

### 2.2. Mineral and Microbial Morphology

#### 2.2.1. Fluorescence Microscopy Results

The mineral residues of *A. niger* and *A. ferrooxidans* and the IREO on day 8 or day 16 were observed under a fluorescence microscope after DAPI staining, and the results are shown in Figure S3. Figure S3a,c shows that fluorescence was generated on the surface of the minerals in the Bio_RE group, which indicates that *A. niger*/*A. ferrooxidans* were adsorbed on the surface of the minerals. As shown in Figure S3b,d, there were only tiny scattered fluorescent spots in the Bio group, indicating that *A. niger*/*A. ferrooxidans* existed in a free state. According to the above results, *A. niger* and *A. ferrooxidans* have similar living states in the microbe–mineral interaction systems and can be roughly divided into two types: those adsorbed on the surface of minerals and those in the form of free bacteria.

#### 2.2.2. Mineral Surface Morphology Characterization via SEM

**Figure 5:**
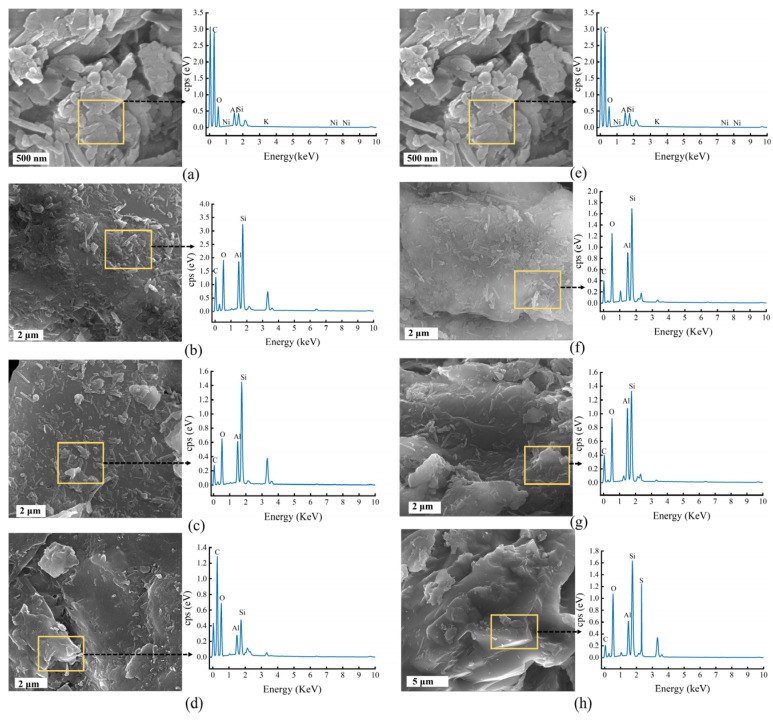
SEM and EDS images of the original (**a**,**e**), Abio_RE (**b**,**f**), Leachate_RE (**c**,**g**), and Bio_RE (**d**,**h**) in the *A. niger* (**b**–**d**)/*A. ferrooxidans* (**f**–**h**)—rare earth ore interaction system.

The mineral residues of *A. niger* and *A. ferrooxidans* and the IREO on day 8 or day 16 were analyzed via SEM and EDS, respectively, and the results are shown in Figure 5. Figure 5a,e shows SEM and EDS images of the original minerals. The IREO was composed of a large number of stacked flake and rod rare earth ores, and the main elements were C, O, Si, and Al. Figure 5b and Figure 5f show SEM and EDS images, respectively, of the mineral residue of the Abio_RE group after the interaction of *A. niger* and *A. ferrooxidans* with the IREO. SEM images revealed that the morphology of the rare earth ore in the Bio_RE group was not significantly different from that of the original rare earth ore; many flake and rod minerals remained on the surface, and the element content did not significantly change. Figure 5c,g shows that, after the interaction of *A. niger* and *A. ferrooxidans* with the ionic rare earth minerals, the surface flake and rod minerals of the mineral residue in the Leathate_RE group decreased significantly, and the surface flake and rod minerals of the mineral residue after the interaction of *A. ferrooxidans* with the ionic rare earth minerals decreased. This may be due to changes in the surface morphology resulting from the redox activity of the metabolites, such as organic acids and extracellular enzymes [23]. In addition, the EDS diagram shows that the contents of Si and Al increased significantly, both of which indicate the dissolution of minerals. Figure 5d,h shows that, after the interaction of *A. niger* and *A. ferrooxidans* with the ionic rare earth minerals, the surface of the mineral residue of the Bio_RE group almost disappeared, and the surface was relatively rough with traces of corrosion. These results suggest that the acid hydrolysis of the two microorganisms was due mainly to the action of H^+^ ions, which separate REEs from minerals through proton exchange, resulting in changes in mineral surface morphology [26]. Among the groups, there were fewer mineral particles on the surface of the mineral residue after the interaction of *A. ferrooxidans* with the ionic rare earth minerals, and the corrosion traces were more severe. The filaments of *A. niger* adsorbed small particles of minerals. These results indicate that *A. niger* and its metabolites can dissolve surface minerals during the leaching of ionic rare earth minerals. In conclusion, *A. niger* filaments can adsorb some small particles of minerals, and *A. ferrooxidans* causes more serious dissolution of minerals.

#### 2.2.3. TEM Results

**Figure 6:**
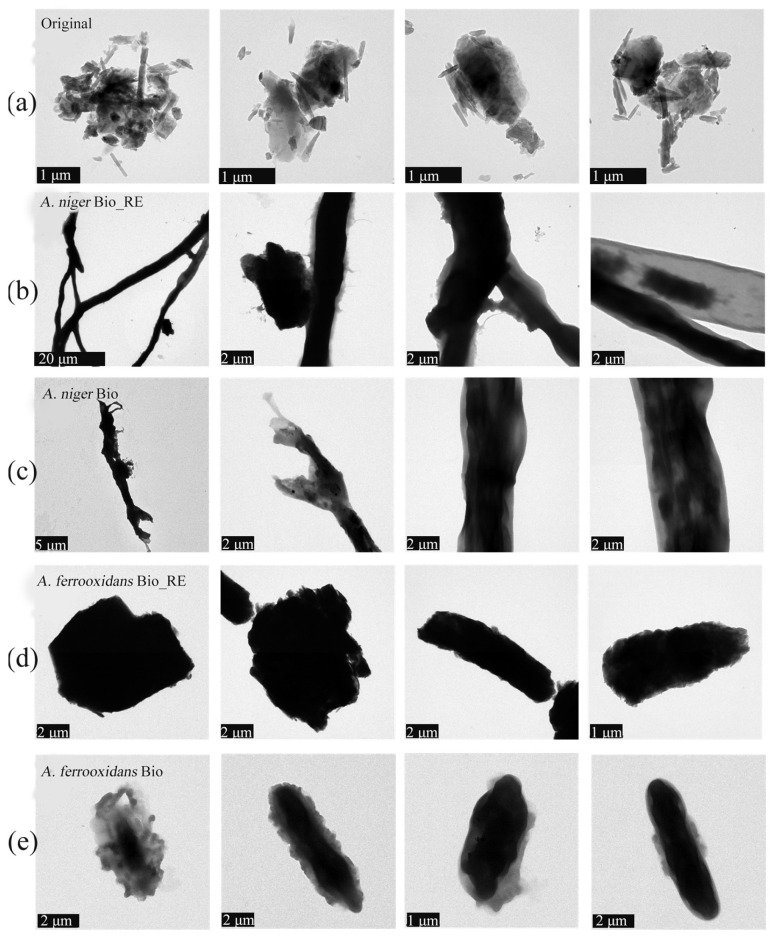
TEM morphology (**a**–**e**) of the original (**a**), Bio_RE (**b**,**d**), and Bio (**c**,**e**) groups in the *A. niger* (**b**,**c**)/*A. ferrooxidans* (**d**,**e**)—rare earth ore interaction system.

**Figure 7:**
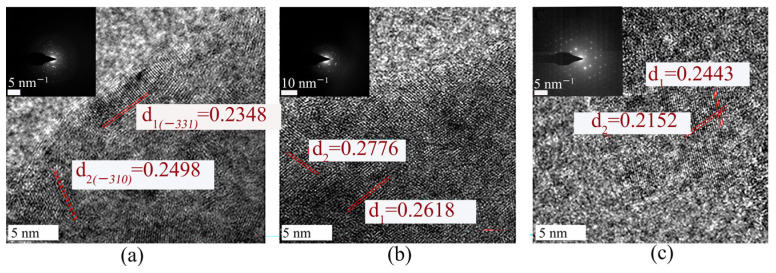
HRTEM images of the original (**a**) and Bio_RE (**b**,**c**) groups in the *A. niger* (**b**)/*A. ferrooxidans* (**c**)—rare earth ore interaction system.

TEM and EDS analyses were conducted for *A. niger* and *A. ferrooxidans* cells and the adsorbed ionic rare earth minerals on day 8 or day 16 (Figure 6 and Figure S4). TEM maps and elemental distribution maps of the original minerals were created (Figure 6a and Figure S4a). Many small rod-shaped particles were present on the surface of the minerals, and many REEs were distributed on the surface of the original minerals. According to Figure 6b,c, small particles of minerals were adsorbed on the surface of the *A. niger* filaments, and the number of rod particles on the surface of the minerals was significantly reduced. The element distribution map of *A. niger* in the Bio_RE group in Figure S4b shows that the number of REEs on the surface of the ionic rare earth minerals was significantly lower than that on the surface of the ionic rare earth minerals, whereas a large amount of REEs was absorbed on the surface of *A. niger*. According to Figure 6d,e, *A. ferrooxidans* was adsorbed on the surface of the IREO, the rod particle minerals on the surface of the IREO almost disappeared, and the surface of *A. ferrooxidans* was rough when rare earth ore was present. The element distribution diagram of *A. ferrooxidans* in the Bio_RE group in Figure S4d shows that the content of REEs on the mineral surface was slightly low, indicating that *A. ferrooxidans* effectively promoted the release of REEs in ionic rare earth minerals. The results revealed that, during the interaction of *A. niger* and *A. ferrooxidans* with rare earth minerals, REEs were partially adsorbed to cells. The content of residual REEs on the surface of rare earth minerals after treatment with *A. niger* was significantly greater than that after treatment with *A. ferrooxidans*, which indicates that the effect of *A. ferrooxidans* on rare earth minerals was greater than that of *A. niger*. Figure 7 shows HRTEM images of the slag before and after the interaction of *A. niger* and *A. ferrooxidans* with ionic rare earth minerals. The results revealed that the lattice streaks of the ionic rare earth minerals significantly changed after microbial leaching, indicating that microorganisms and their metabolites led to structural changes in the ionic rare earth minerals.

#### 2.2.4. Mineral-Phase Transformation

**Figure 8:**
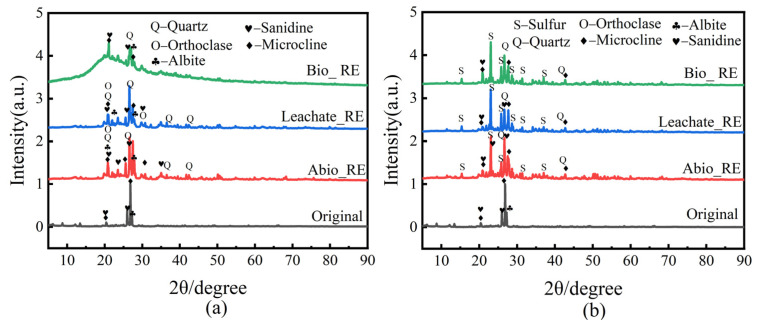
XRD patterns of primitive rare earth ore and 8 d slag in the interaction system of *A. niger* (**a**)/*A. ferrooxidans* (**b**).

XRD analysis was conducted on the minerals before and after the interaction of *A. niger* and *A. ferrooxidans* with the ionic rare earth minerals, and the results are shown in Figure 8. The main components of the original rare earth mineral phase were microcline, sanidine, and xanthite. After the interaction between *A. niger* and the IREO, a large amount of quartz appeared in the rare earth ore residue of the Abio_RE group. The residue of the Leachate_RE group of *A. niger* was mainly composed of quartz, sanidine, microcline, yellow feldspar, and orthoclase. Compared to that of the original minerals, the content of microcline was lower, and the contents of phases such as quartz and orthoclase were greater. The leaching residue of IREO in the Bio_RE group of *A. niger* was mainly composed of quartz, sanidine, yellow feldspar, and microcline (Figure 8a). After the interaction between *A. ferrooxidans* and the IREO, a large amount of quartz appeared in the rare earth ore residue (Figure 8b). Compared to that of the original ore, the diffraction peak of the leached ore did not change significantly, indicating that the ore structure was not damaged, which may be conducive to the stability of the ore body and is crucial for in situ bioleaching [33]. The results revealed that the mineral residue underwent dissolution and phase transformation after the interaction between *A. niger* and the IREO, whereas the ore structure did not change significantly after the interaction between *A. ferrooxidans* and the IREO.

#### 2.2.5. Mineral and Microbial Surface Functional Groups

**Figure 9:**
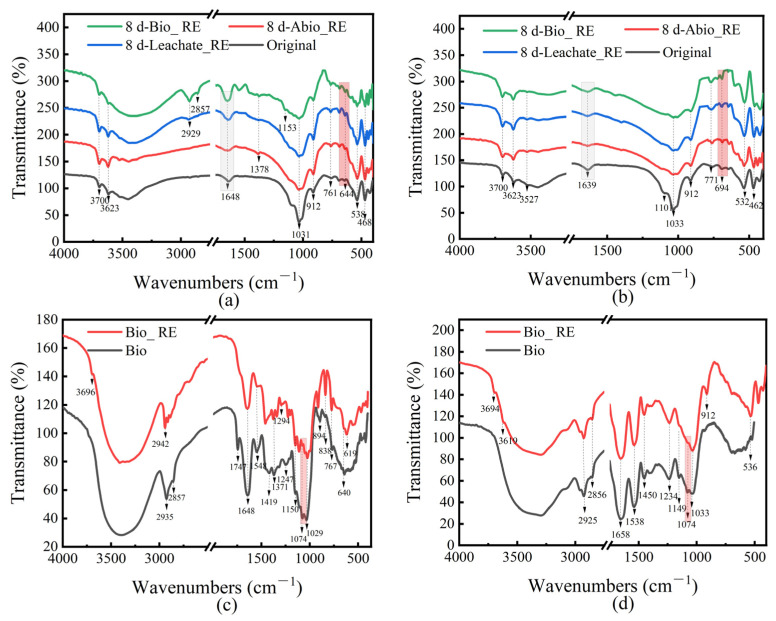
FTIR spectra of Abio_RE, Leachate_RE, and Bio_RE in the *A. niger* (**a**)/*A. ferrooxidans* (**b**)—rare earth ore interaction system on the original and 8th/16th days, and FTIR diagram of cells in the Bio_ RE and Bio in the *A. niger* (**c**)/*A. ferrooxidans* (**d**) interaction system on day 8/day 16.

FTIR analysis was performed on the minerals before and after the interaction between *A. niger* and *A. ferrooxidans* and ionic rare earth minerals, and the results are shown in Figure 9. The infrared spectra of the IREO residues, especially those in the Bio_RE group, changed significantly after leaching. After bioleaching, there was a slight shift in the peak at approximately 1640 cm^−1^, which was attributed to the C=O stretching vibration, confirming that the carboxylic group was generated and involved in leaching [26]. Vibration peaks were found at 694 and 644 cm^−1^, indicating the presence of feldspar and quartz. Most of the peak spectra before and after leaching were similar, which may indicate that the main mechanism was acid hydrolysis and complexation. Owing to the biochemical characteristics of *A. ferrooxidans*, the bacterium can use O_2_ as an electron acceptor to oxidize elemental sulfur and reduce inorganic sulfur compounds to sulfate, thus promoting the release of REEs in ionic rare earth minerals through acid hydrolysis [34].

Figure 9c,d shows the FTIR spectra of cells in Bio_ RE and Bio on day 8/day 16 in the system of interactions of *A. niger* and *A. ferrooxidans* with IREOs. The vibration of the peak at 1074 cm^−1^ also indicates the participation of C−OH [35]. FTIR analysis revealed that hydroxyl, amine, and carboxyl groups from polysaccharides and proteins may be involved in the bioleaching of REEs by *A. niger* and *A. ferrooxidans*.

### 2.3. Comparative Transcriptomics

**Figure 10:**
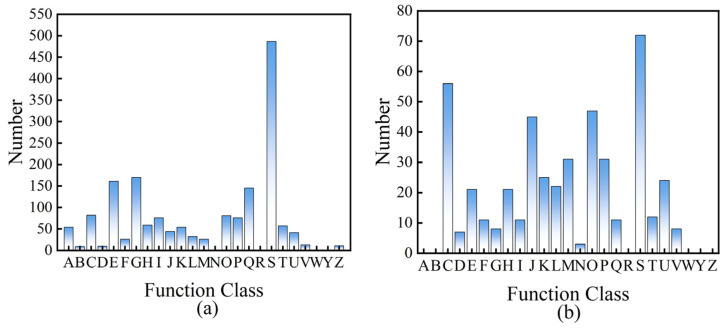
Functional classification of eggNOG consensus sequences for *A. niger* (**a**) and *A. ferrooxidans* (**b**). A: processing and modification of RNA; B: chromatin dynamics and structure; C: production and conversion of energy; D: control of the cell cycle, cell division, and chromosome distribution; E: transport and metabolism of AAs; F: transport and metabolism of nucleotides; G: transport and metabolism of carbohydrates; H: transport and metabolism of coenzymes; I: transport and metabolism of lipids; J: translation, ribosomal architecture, and biogenesis; K: transcription; L: replication mechanisms, recombination, and repair pathways; M: biogenesis of cell walls, membranes, and envelopes; N: cell motility; O: protein turnover, posttranslational modification, and chaperone functions; P: transport and metabolism of inorganic ions; Q: biosynthesis, transport, and catabolism of secondary metabolites; R: general function prediction only; S: function unknown; T: with regard to signal transduction mechanisms; U: intracellular trafficking, secretion, and vesicular transport; V: defense mechanisms; W: extracellular structures; Y: nuclear structure; Z: cytoskeleton.

We conducted transcriptome analyses of the bacteria before and after the interactions between *A. niger* and *A. ferrooxidans* and the IREO, and the results are shown in Figure 10 and Figure S5. Analysis of the differentially expressed genes (DEGs) of *A. ferrooxidans* before and after interaction with IREO revealed that the DEGs of the two strains tended to decrease in the presence of the rare earth ore.

A comparison of the metabolic pathways associated with the upregulated and downregulated genes revealed that the presence of IREO can promote the growth and division of *A. ferrooxidans*. In addition, the presence of rare earth minerals has a positive effect on the transport and metabolism of nucleotides and coenzymes and the transcriptional posttranslational modification of *A. ferrooxidans*, i.e., transport, protein synthesis, the biosynthesis of secondary metabolites, and catabolism. However, the presence of rare earth minerals can inhibit the transport and metabolism of amino acids (AAs), translation processes, ribosome structure and biogenesis, cell wall and cell membrane biosynthesis, cell modification, inorganic ion transport and metabolism, intracellular biological transport and defense mechanisms, and other biological processes.

In addition, according to the Kyoto Encyclopedia of Genes and Genomes (KEGG) pathway analysis, the *gpmL*, *gpmB*, *apgM*, *PK*, *PKLR*, *gdh,* and *gmd* genes of *A. ferrooxidans* are significantly upregulated in the presence of rare earth minerals, which can promote the secretion of sugars and the synthesis of AAs. The *UbiC* and *XanB2* genes are upregulated to promote the biosynthesis of ubiquinone and other terpenoid quinones. Ubiquinone plays a vital role in the electron transport chain of microorganisms. During the interaction of *A. ferrooxidans* with rare earth ores, the electron transport chain is essential for bacteria to obtain energy through redox reactions. The *UbiC* gene helps synthesize ubiquinone, which guarantees the proper function of the electron transport chain, allowing bacteria to efficiently use energy sources such as ferrous iron and sulfur in rare earth ores and convert them into chemical energy for cells [36]. *FabF*, *FASN*, *FAS2,* and *PLX* genes were significantly downregulated, which inhibited lipid biosynthesis and metabolism, thus inhibiting the adsorption of microorganisms and the surface of rare earth ores [37]. *MoaC* gene downregulation inhibited sulfur reduction. The related genes encoding cytochrome C were downregulated, and the transmission of respiratory chain electrons was inhibited. There was no significant effect on organic acid secretion during the tricarboxylic acid cycle. In the presence of rare earth minerals, the ALDH genes of *A. niger* were significantly upregulated in the pentose phosphate pathway and promoted the uronic acid pathway. *pfkA*, *PFK*, *GAPDH*, *gapA*, *PGK*, *PDC*, *gpmI*, *gnd*, *gntZ,* and other genes inhibited the glycolysis pathway, thus inhibiting the formation of adenosine triphosphate. In the citric acid cycle, the ACO and *acnA* genes were upregulated to promote the biosynthesis of cis-aconitic acid and citric acid. *LSC1* and other genes were significantly downregulated, which inhibited the biosynthesis and metabolism of succinic acid and the succinic acid coenzyme A. The results revealed that the presence of ionic rare earth minerals can promote the growth and metabolism of *A. ferrooxidans* but inhibit the biofilm and fatty acid synthesis of *A. ferrooxidans*, whereas it can promote the tricarboxylic acid cycle of *A. niger*.

### 2.4. Overall Metabolomic Profile

**Figure 11:**
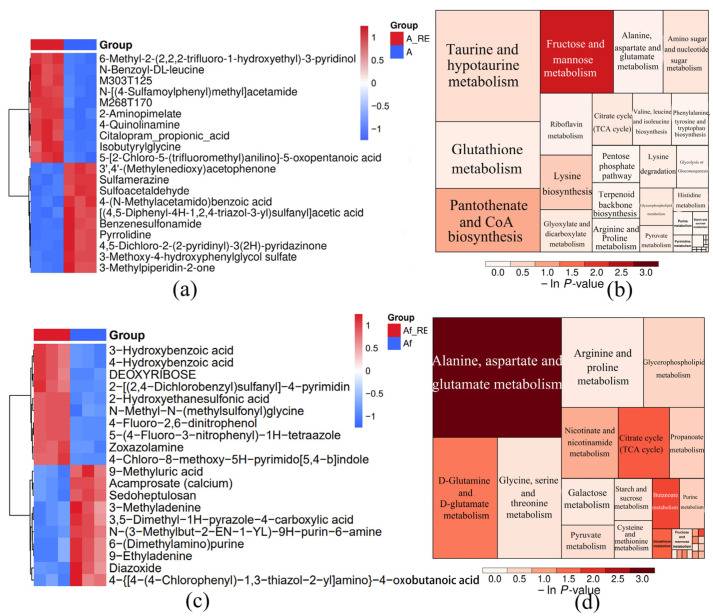
Metabolomic statistical analysis of *A. niger* (**a**,**b**) and *A. ferrooxidans* (**c**,**d**). (**a**,**c**): clustering heatmaps of the top 10 differentially abundant metabolites; (**b**,**d**): KEGG tree map of the differentially abundant metabolite aggregation.

Under the previously mentioned experimental conditions, culture supernatants of *A. niger*/*A. ferrooxidans* supplemented with IREO were collected for nontargeted metabolomic analysis at days 8 and 16, as shown in Figure 11.

Clustering techniques were employed to simplify datasets by grouping similar data points. Additionally, PLS-DA within PCA was utilized to evaluate the capacity to discern distinct metabolic profiles. PCA (Figures S6a and S7a) and PLS-DA (Figures S6b,c and S7b,c) revealed significant differences. The volcano plot shows a significant difference between the two groups (regardless of whether they contained metabolites of rare earth minerals) (Figures S6d and S7d). The parameters Q2 and R2Y of the PLS model can be used to evaluate the prediction and modeling capabilities in OPLS analysis, respectively (Table S1). Higher cumulative values approaching unity reflect greater model stability and reliability. In this study, the OPLS-DA model demonstrated parameter values near 1, suggesting the establishment of a statistically valid and interpretable analytical framework. These results confirm the model’s robustness and suitability for meaningful data interpretation within the experimental context.

The metabolomic results of the *A. niger* culture medium revealed that there were significant differences in 756 metabolites before and after leaching, of which 183 metabolites were upregulated, and 573 metabolites were downregulated, as shown in Figure S6d. The metabolite classification is shown in Figure S6f. A significant portion of the downregulated metabolites (15.88%, Table S2) consisted of organic acids and their related derivatives, whereas lipids and lipid-like compounds represented 5.46% of the upregulated metabolites. This distribution highlights the distinct compositional shifts in metabolic profiles, with organic acids dominating the downregulated category and lipid-based molecules contributing notably to the upregulated group. The KEGG annotation of the metabolic pathways of the differentially abundant metabolites revealed that the metabolites were associated mainly with the metabolism of AAs, carbon, lipids, nucleotides, and energy and the ATP-binding-cassette (ABC) transporters (Figure S6f). According to the cluster heatmap analysis, the main upregulated metabolites were 6-methyl-2-(2,2,2-trifluoro-1-hydroxyethyl)-3-pyridinyl, N-benzoyl-DL-leucine, N-[(4-sulfamoyl phenyl)methyl]acetamide, 2-aminopyridine, citalopram_propionic acid, isobutyrylglycine, 5-[2-chloro-5-(trifluoromethyl)aniline]-5-oxovaleric acid, etc. The main downregulated metabolites were 3′,4′-(methylenedioxy)acetophenone, sulfonamidesine, sulfoacetaldehyde, 4-(N-methylacetamido)benzoic acid, [(4,5-diphenyl-4H-1,2,4-triazol-3-yl)sulfonyl]acetic acid, pyrrolidine, 4,5-dichloro-2-(2-pyridyl)-3(2H)-pyridazine, 3-methoxy-4-hydroxyphenyl glycol sulfate, and 3-methylpiperidin-2-one. Based on the main differentially abundant metabolites, 50 different metabolites were grouped into specific classes, as shown in Tables S2–S6. Twelve of the 15 metabolites of organic acids and their derivatives were downregulated. The main downregulated AAs were proline, betaine, N-acetylhistamine, γ-aminobutyric acid, L-yeastine, glutamic acid, trans-aconitic acid, malic acid, (2S)-2-hydroxyglutaric acid, ethyl acetoacetate, cis-aconitic acid, and 2-ketoglutarate.

Previous studies have demonstrated that *A. niger* predominantly synthesizes several key organic acid metabolites—citrate, gluconic, malate, and succinate. However, certain organic acids are not directly identifiable, probably because their presence is in derivative forms, such as 2-methyl citric acid and D-glucuronic acid. These observations highlight the influence of organic acids on sample analysis and extraction efficiency. Comparative analysis of microbial metabolites before and after extraction revealed significant alterations, indicating active interactions between the metabolites and rare earth ores. Furthermore, the evaluation of various product types revealed a substantial proportion of organic acids and their derivatives, underscoring their dominant role in the metabolic profile. The metabolites at this ratio were mainly downregulated, and their effects were relatively large compared to those of various metabolites in the leaching process [30].

The metabolomic results of the *A. ferrooxidans* culture medium revealed significant differences in 120 metabolites before and after leaching, of which 52 metabolites were upregulated and 68 metabolites were downregulated, as shown in Figure S7d. The metabolite classification is shown in Figure S7e. The proportions of organic acids and their derivatives and of lipids and lipid-like molecules among the downregulated metabolites of *A. ferrooxidans* were 11.76% and 25%, respectively, which are very different from those of *A. niger* (Table S2). The metabolic pathways associated with the differentially abundant metabolites of *A. ferrooxidans* were similar to those associated with *A. niger*, as shown in Figure 11d. The main upregulated metabolites were 3-hydroxybenzoic acid, 4-hydroxybenzoic acid, deoxyribose, 2-[(2,4-dichloro benzyl)sulfonyl]−4-pyrimidine, 2-hydroxyethanesulfonic acid, N-methyl-N-(methylsulfonyl)glycine, 4-fluoro-2,6-dinitrophenol, etc. The main downregulated metabolites were 9-methyluric acid, 3-methyladenine, 3,5-dimethyl-1H-pyrazole-4-carboxylic acid, N-(3-methyl-2-EN-1-yl)-9H-purine-6-amine, 6-(dimethylamino)purine, 9-ethyladenine, diazoxide, etc. (Figure 11c). Among the top 50 differentially abundant metabolites, organic acids and lipids and their derivatives accounted for 16% and 22%, respectively. Among them, four of the organic acids and their derivatives were upregulated, and three of the lipid and lipid-like molecules were upregulated. In the case of the IREOs, the metabolites tended to decrease, indicating that they had an important impact on their leaching.

## 3. Discussion

**Figure 12:**
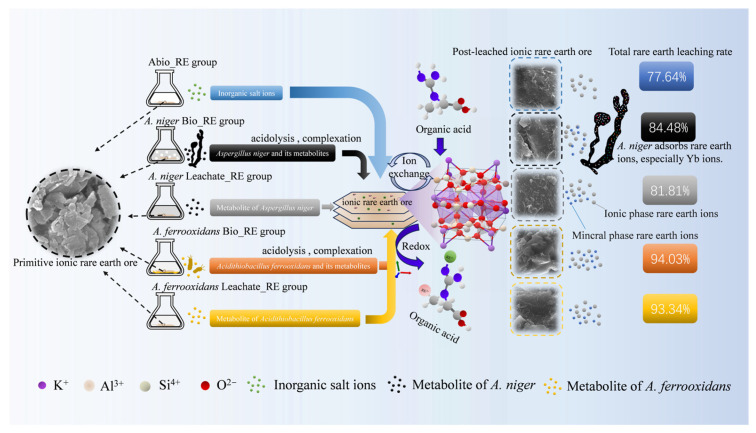
Diagram of the leaching mechanisms of IREO by *A. niger* and *A. ferrooxidans*.

This study revealed that *A. niger* and *A. ferrooxidans* play important roles in the biological extraction of IREOs, and it investigated the complex mechanism of microbial metabolites in the leaching of REEs. By observing the changes in the concentration of REEs in the solution with these two microorganisms, the leaching of rare earth ions was significantly affected by a variety of factors, including the cellular concentration, metabolite concentration, and solution pH. Taken together, these factors determine the efficiency of the release of rare earth ions. Notably, in the absence of microorganisms, the experimental results of the Abio group revealed that, although the inorganic salt ions in the medium can effectively leach most of the ionic REEs, the leaching effect of mineral-phase REEs was relatively poor. This underscores the importance of microorganisms in the extraction process, as they significantly increased the release of REEs from the mineral phase through a unique metabolic mechanism, as shown in Figure 12.

During leaching experiments with *A. niger* and *A. ferrooxidans*, their metabolites, such as organic acids, significantly reduced the pH of the solution. This pH reduction promoted the destruction of the surface structure of the ore, thereby promoting the liberation of REEs in the mineral phase. The acidic metabolites used to enhance the leaching effect interacted with mineral phases and rare earth ions through chemical pathways to form more soluble complexes. The SEM results indicated that, after the interaction with *A. niger* and *A. ferrooxidans*, the surface of the mineral residue was rougher, and there were obvious traces of corrosion on the surface. Moreover, the number of small mineral particles decreased significantly, indicating that organic acids, AAs, and other microbial metabolites largely damaged the surface structures of the IREOs and promoted the release of ions.

In addition to the above effects, the reducing components in the microbial metabolites may also be involved in the change in the surface morphology of the ore. We hypothesized that the resulting extracellular enzymes, such as oxidases [38], react with the mineral surface and contribute to further deconstruction of the mineral matrix. Moreover, the results of XRD and FTIR spectroscopy revealed that the phase and peak spectra of the minerals before and after leaching remained relatively consistent. This finding suggests that the main mechanisms of leaching are acidolysis and complexation and that the characteristic peaks of organic acids in the infrared spectrum are evidence of metabolite involvement.

A comparison of the results of transcriptomic and metabolomic studies revealed that, when rare earth minerals are present, the genes encoding lipids and organic acids significantly differ. This phenomenon strongly suggests that microbial responses to rare earth minerals are not limited to physical and chemical actions but also involve changes at the gene expression level. The metabolomic results revealed changes in microbial metabolites before and after leaching, suggesting that these metabolites may have complex interactions with IREOs. In addition, the analysis of different metabolites revealed that organic acids and their derivatives play important roles in the whole process, and most of these products tended to decrease. These results suggest that these organic acids are relatively important in the REE leaching process and that their role may be closely related to the hydrolysis and complexation of rare earth ions.

The core of the acidolysis mechanism is that microbial metabolites lower the pH by releasing protons into the leaching solution, thereby promoting the supply of metals and anions to minerals. Changes in the metal and anion concentrations affect the formation of certain rare earth ions and complexes [39]. For example, gluconic acid produced by *A. niger* and *A. ferrooxidans* may effectively dissolve the mineral matrix through an acid cleavage mechanism, accelerating the release process of REEs [40]. The hydroxyl, carboxyl, and other functional groups in the differentially abundant metabolites may also be involved in metabolic pathways. The structural characteristics of these functional groups allow them to effectively form stable complexes with rare earth ions, further promoting the leaching of REEs.

Finally, in ionic rare earth minerals, microbial leaching has unique advantages [8]. Compared to traditional chemical leaching, it lowers costs and limits pollution, which is conducive to the green leaching of IREOs. *A. niger* mainly secretes organic acids such as citric acid and undergoes acidolysis and complexation reactions with rare earth ions in the form of ionic bonds and coordination bonds to achieve the noncontact leaching of REEs [26]. *A. ferrooxidans*, which relies mainly on biological oxidation, creates a favorable redox potential and pH environment for rare earth leaching and uses its acid-producing characteristics to form a low pH environment to dissolve rare earth minerals [41]. Chemical reagents typically promote the release of rare earth ions from minerals through ion exchange [42]. Organic acids can compete with rare earth ions to reduce the Gibbs free energy of rare earth leaching reactions, whereas some organic acid systems have a buffering capacity to maintain a certain pH environment, thereby promoting the dissociation of rare earth ions from minerals [43]. Chemical leaching agents, including ammonium sulfate, often result in significant environmental contamination. In contrast, certain organic acids exhibit superior biocompatibility and generate a comparatively lower environmental impact than conventional strong acids and other extraction agents. Nevertheless, the expenses associated with the synthesis and acquisition of organic acids differ based on their specific types; some may incur high costs, and additional supplementary reagents might be required during the extraction process to enhance efficiency, potentially escalating overall expenses. In microbial extraction approaches, the expenditure related to microbial cultivation and fermentation is relatively modest and can be further minimized through process optimization; the overall leaching cost has certain advantages. The waste generated in the leaching process is mainly organic acid residue and biomass residue, and the organic acid residue can be self-degraded through neutralization treatment and biomass, with little impact on the environment [44].

In the future, by improving environmental protection requirements, the leaching technology of *A. niger* and *A. ferrooxidans* will be greener and more sustainable. Through the refinement of microbial cultivation parameters and the advancement of hybrid extraction solutions coupled with effluent management systems, extraction performance can be enhanced while minimizing ecological impact. This approach not only improves process efficacy but also contributes to sustainable practices by reducing environmental contamination. It will be possible to combine genetic engineering, metabolic engineering, and other technologies to modify microorganisms to improve their leaching ability. Furthermore, the implementation of smart and automated extraction systems has been introduced to lower operational expenses and enhance the practicality of large-scale industrial utilization. These innovations streamline production processes while increasing cost-efficiency and scalability for industrial adoption.

## 4. Materials and Methods

### 4.1. Materials

The IREO used in the experiments was taken from the Jianghua rare earth mining area, dried, and ground into powder IREO samples, and samples with particle sizes of 38~74 μm were taken for the experiments. Before the experiment, XRD was used to analyze the phase composition of the ore sample, as shown in Figure S8a. The results show that the sample phase of the IREO used in this experiment was mainly composed of microcline, sanidine, and melilite, which are relatively classical IREOs. X-ray fluorescence spectroscopy and inductively coupled plasma mass spectrometry were used to detect the compounds and REEs of the IREO. The results are shown in Table S7 and Figure S8, and the total rare earth oxide content was approximately 0.52%.

The strains of *A. niger* (No. GDMCC 3.516) and *A. ferrooxidans* (No. ATCC 23270) were purchased from Guangdong Microbial Culture Preservation Center (GDMCC) and provided by the Key Laboratory of Biometallurgy of the Ministry of Education, School of Minerals Processing and Bioengineering, Central South University. PDA medium, fermentation medium, and 9K basic medium were used to activate the experimental strains, and the culture was expanded until it reached the stable period of bacterial harvest, while the fermentation medium was preserved for use. The related components of the medium are shown in Table S8.

### 4.2. Method

*A. niger* was activated in PDA media and cultured at 37 °C for approximately 5 days. Then, 5–10 mL of 0.05% Tween 80 in normal saline solution was added, and the surface impurities were washed to better disperse them in the aqueous solution. *A. niger* conidiospores were scraped into the solution, and the resulting bacterial suspension was diluted to 107 by viable bacteria counting, after which an *A. niger* spore suspension was obtained for use.

One hundred milliliters of fermentation medium or 9K medium was placed in a 250 mL conical bottle. After sterilization, a 1 g IREO sample was added, and the *A. niger* spore suspension, *A. ferrooxidans,* and fermentation mixture were added. The interaction system was set up as follows: In the *A. niger*/*A. ferrooxidans*–ionic rare earth direct interaction system (Bio_RE group for short), the microbial inoculum volume was 1 × 10^7^ /mL; the fermentation broth of *A. niger* and *A. ferrooxidans*, cultured to a stable stage, was added to the indirect system (Leachate_RE group for short). All the experimental systems were cultured in a fully temperature-controlled shaker (ZQZY-A8) at 37 °C (*A. niger*) and 30 °C (*A. ferrooxidans*) with a pH value of 2.0 ± 0.2 and at 180 r/min vibration for 8 or 16 days. The sterile experimental group containing only pure medium (Abio group), the experimental group containing only ionized rare earth minerals (Abio_RE group), and the experimental group containing only microorganisms in the medium (Bio group) were used as controls. All the experimental systems were set up in triplicate.

### 4.3. Analytical Methods

Liquid- and mineral-phase samples were taken at 1 d intervals. For liquid samples, the cell concentration was measured directly under an optical microscope (CX33, Olympus, Tokyo Metropolis, Japan) via a blood cell counting plate. The samples in the Bio-Re group and the Bio group were dyed with nucleic acid dye 4′, 6-diaminyl-2-phenylindole (DAPI) and observed via a fluorescence microscope (Nexcope NE900, Ningbo Yongxin Instrument Co., Ltd., Ningbo, China). The combination of DAPI and DNA produces blue-purple fluorescence under a fluorescence microscope. A pH meter (FE28, Mettler Toledo International Co., Ltd., Shanghai, China) and glass pH electrode potential measurement were used to determine the pH, Ag/AgCl as the reference electrode was used to determine the redox potential (ORP), an inductively coupled plasma emission spectrometer (ICP-OES; IRIS Intrepid 161 II XSP, Thermo Fisher, USA) was used to detect the concentration of REEs in the solution, and TEM (Talos F200X, Thermo Fisher, Waltham, MA, USA) was used to characterize the cell morphology before and after leaching. The cell surface functional groups were determined via FTIR (Nexus 670, Nicolet, Waltham, MA, USA). For the solid samples, fluorescence microscopy was used to observe the adsorption of the cells and ionized rare earth minerals, and SEM-EDS (SEM; MIRA4 LMH, EDS; Oxford AZtecLive Ultim Max 20, Oxford, UK) was used to observe the mineral morphology and element composition. TEM (Talos F200X, Thermo Fisher, Waltham, MA, USA) was used to characterize the distribution of bacteria adsorbed on the surface of the minerals, and FTIR (Nexus 670, Nicolet, Waltham, MA, USA) was used to determine the surface functional groups of the minerals. Global metabolomic profiling was conducted using liquid chromatography coupled with high-resolution mass spectrometry (LC-MS). Metabolites exhibiting significant changes were identified based on variable importance in projection (VIP) scores derived from the OPLS-DA model and statistical significance from Student’s *t*-test (VIP > 1, *p* < 0.05). A clustered heatmap was generated to visualize the patterns of these altered metabolites. The Kyoto Encyclopedia of Genes and Genomes (KEGG) database was utilized to annotate the metabolic pathways of differentially expressed metabolites and to determine the biological pathways linked to these variations. This approach enabled the systematic mapping of metabolic flux and the identification of relevant biochemical routes associated with the observed changes in metabolite abundance. Metabolomic experiments were performed six times for data collection and processing. In transcriptomic comparative analysis conducted via the BGI platform, gene expression levels were quantified using RSEM, while differential gene expression was evaluated employing the DESeq2 computational tool. This methodological approach facilitated the systematic comparison and identification of transcriptional variations across the studied conditions. After the sequenced genes were compared with the GO and KEGG databases, gene functional classification analysis and annotation of the differential gene sets were carried out, and the GO enrichment and KEGG enrichment results were analyzed by ClusterProfiler software (version 4.6.0) after the functional annotation was completed.

## 5. Conclusions

We studied the interaction mechanism between two typical microorganisms, *A. niger* and *A. ferrooxidans*, and the bioleaching process of IREOs under acidic conditions. The presence of microorganisms was conducive to the leaching of REEs, among which the mycelia of *A. niger* strongly adsorbed Yb, *A. ferrooxidans* promoted the release of REEs more effectively than *A. niger*, and the maximum leaching rate of the latter reached 94.03%. The mineral residue was significantly dissolved and transformed after the interaction between *A. niger* and the IREO, whereas the mineral structure of *A. ferrooxidans* did not change significantly after the interaction with the IREOs, indicating that there were differences in the interactions between the two microorganisms and ionic rare earth minerals. *A. niger* promoted the release of REEs from ionic rare earth minerals by secreting organic acids and other metabolites containing carboxyl functional groups. Owing to its ability to complex with rare earth ions, the carboxyl functional group reacts with rare earth ions on the surface of ionic rare earth minerals to disrupt the original mineral structure, releasing REEs, and *A. ferrooxidans* promotes the release of REEs from rare earth minerals through acidification and complexation. The significant differential expression of metabolic genes and metabolites related to the secretion of organic acids and their derivatives, lipids, and other metabolites indicates their correlation with the leaching process.

## Data Availability

The original contributions presented in this study are included in the article/Supplementary Material. Further inquiries can be directed to the corresponding author.

## Supplementary Materials

The supporting information can be downloaded at: https://www.mdpi.com/article/10.3390/ijms26051986/s1.
