# Supplementary material for: Rare Earth Element Extraction from Ionic Rare Earth Ores by Two Typical Acidogenic Microorganisms, Aspergillus niger and Acidithiobacillus ferrooxidans
Source: Int J Mol Sci. 2025 Feb 25;26(5):1986. doi: 10.3390/ijms26051986 (PMC11900449; doi:10.3390/ijms26051986)
Supplement: Supplementary file 1 [file ijms-26-01986-s001.zip › ijms-3487229-supplementary.pdf]

# Supplementary information

# for

# REE extraction from ionic rare earth ores by two typical acidogenic microorganisms *Aspergillus niger* and *Acidithiobacillus ferrooxidans*

**Mengyuan Wang [a,#], Jinna Li [a,#], Hongchang Liu [a,b,*], Shiyun Huang [a], Xiaoyan Liu [a], Yang Liu [a,b], Muhammad Awais [a], Jun Wang [a,b]**

**Affiliations:**

[a] School of Minerals Processing and Bioengineering, Central South University, Changsha 410083, China.; mengyuanwang@csu.edu.cn (M.W.); li_jingna@csu.edu.cn (J.L.); hchliu2050@csu.edu.cn (H.L.); hsy2023@csu.edu.cn (S.H.); 2079833454@qq.com (X.L.); liuyang_feiyang@163.com (Y.L.); muhammadawaissagar786@gmail.com (M.A.); wjwq2000@126.com (J.W.)

[b] Key Lab of Biometallurgy of Ministry of Education of China, Central South University, Changsha 410083, China

[#] These people contribute equally to this work

[*] Corresponding author: hchliu2050@csu.edu.cn (H.L.)

The part contains eight supplementary figures and eight supplementary tables.

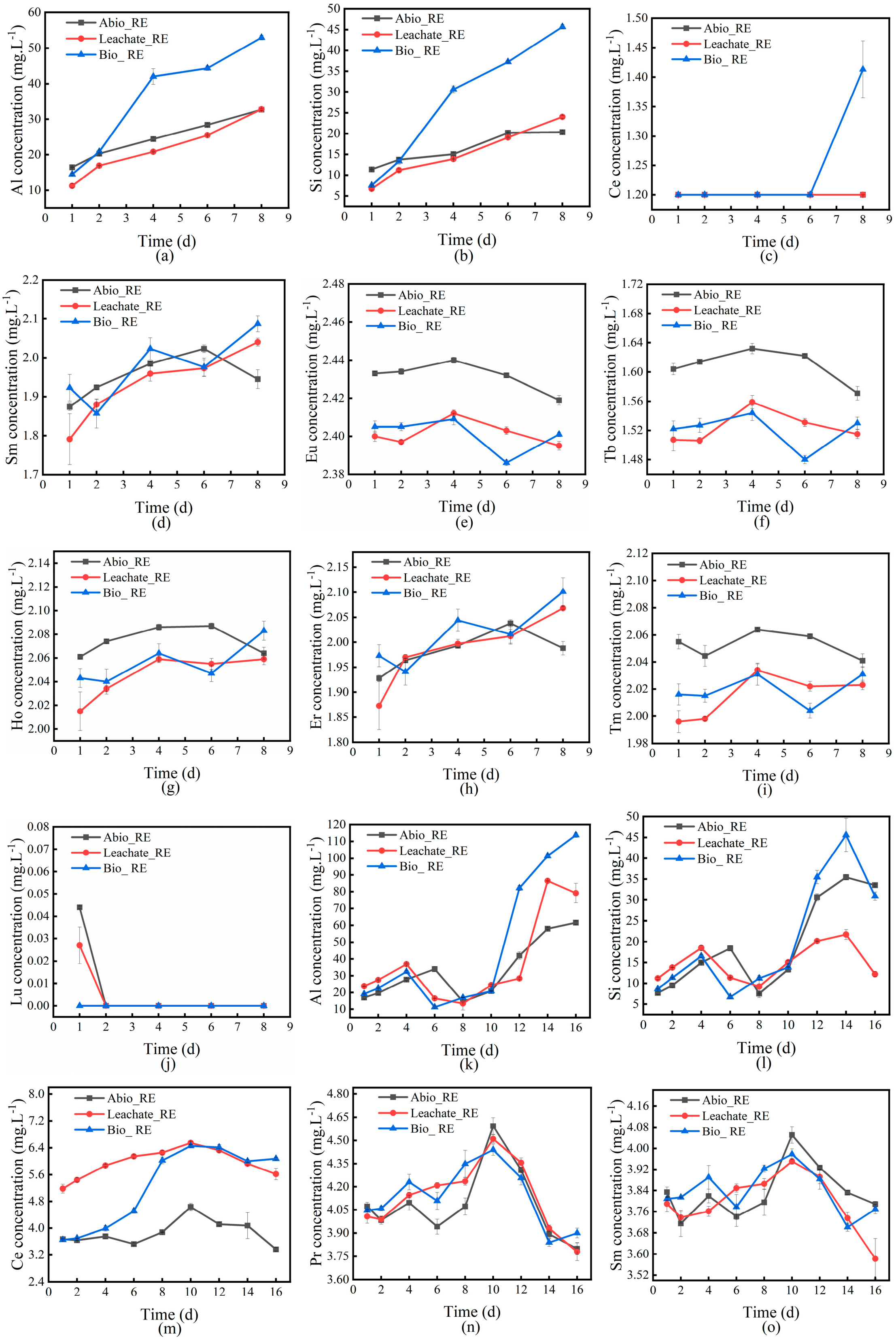

Abio_RE
Leachate_RE
Bio_ RE
Al concentration (mg.L-1)
Si concentration (mg.L-1)
Ce concentration (mg.L-1)
Sm concentration (mg.L-1)
Eu concentration (mg.L-1)
Tb concentration (mg.L-1)
Ho concentration (mg.L-1)
Er concentration (mg.L-1)
Tm concentration (mg.L-1)
Lu concentration (mg.L-1)
Al concentration (mg.L-1)
Si concentration (mg.L-1)
Ce concentration (mg.L-1)
Pr concentration (mg.L-1)
Sm concentration (mg.L-1)
Time (d)
(a)
(b)
(c)
(d)
(e)
(f)
(g)
(h)
(i)
(j)
(k)
(l)
(m)
(n)
(o)

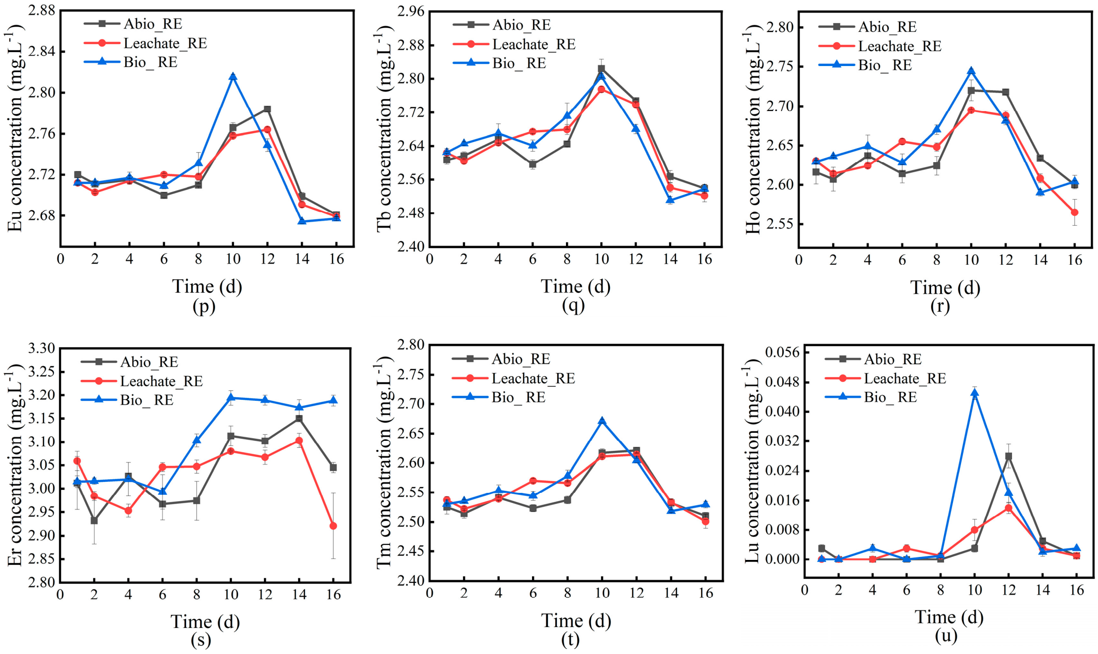


**Figure S1** Concentration curves of Al (a, k), Si (b, l), Ce (c, m), Pr (n), Sm (d, o), Eu (e, p), Tb (f, q), Ho (g, r), Er (h, s), Tm (i, t), and Lu (j, u), in the solution of *A. niger* (a-j)/*A. ferrooxidans* (k-u) - rare earth ore interaction system.

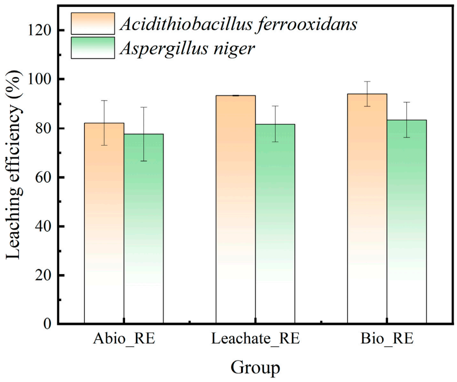


**Figure S2** *A. niger*/*A. ferrooxidans* interact with ionic rare earth minerals and affect the total rare earth leaching rate.

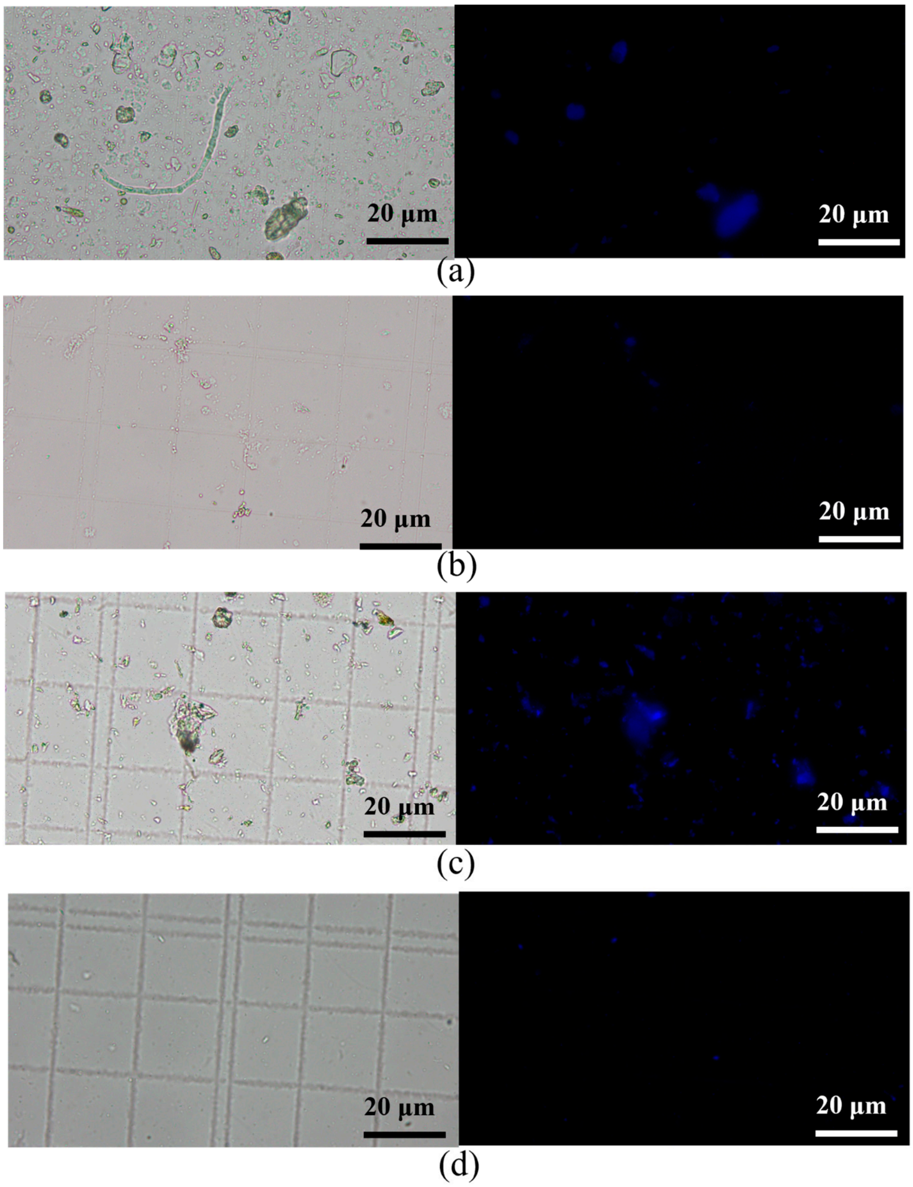


**Figure S3** Microscopically original and fluorescence images of Bio_RE (a, c) and Bio (b, d) on the 8th/16th day in the *A. niger* (a-b)/ *A. ferrooxidans* (c-d) -rare earth ore interaction system.

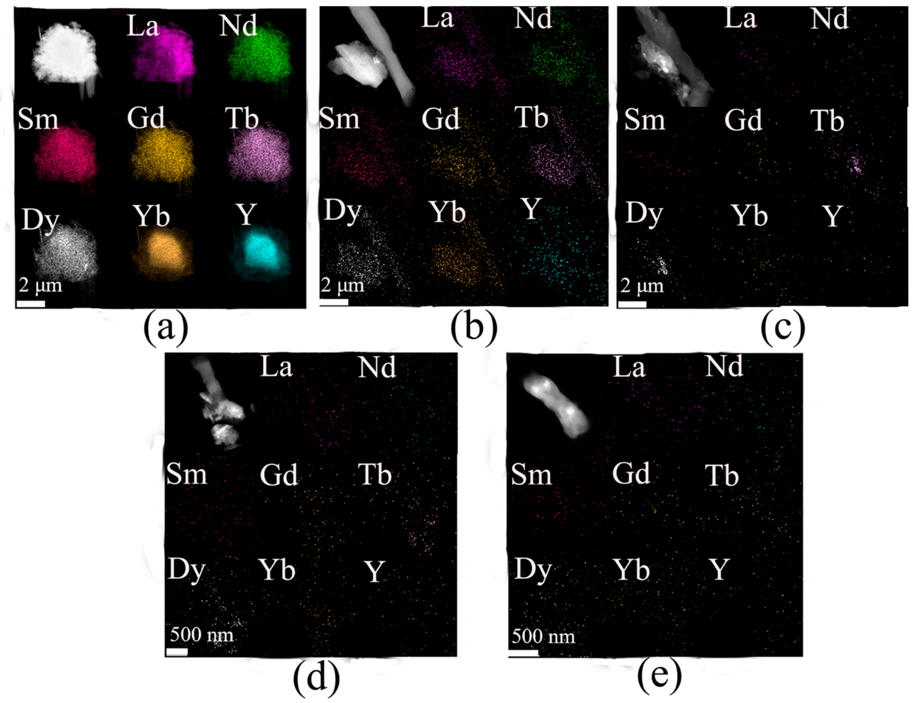


**Figure S4** TEM elemental distribution map (a-e) of the Original (a), Bio_RE (b, d), and Bio (c, e) groups in *A. niger* (b, c)/*A. ferrooxidans* (d, e)-rare earth ore interaction system.

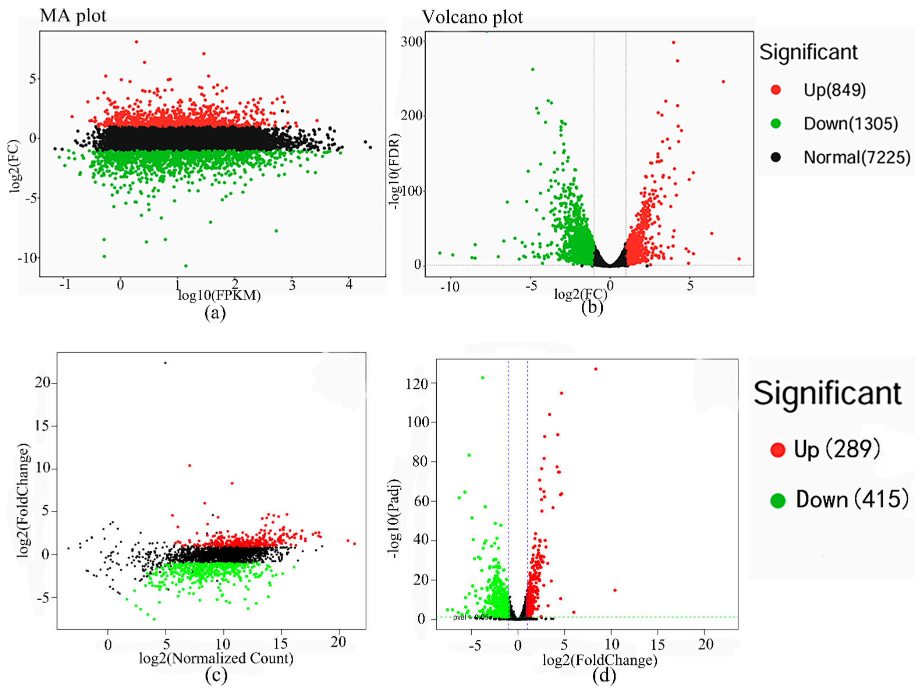


**Figure S5** Comparative transcriptome of *A. niger* (a,b)/*A. ferrooxidans* (c,d).

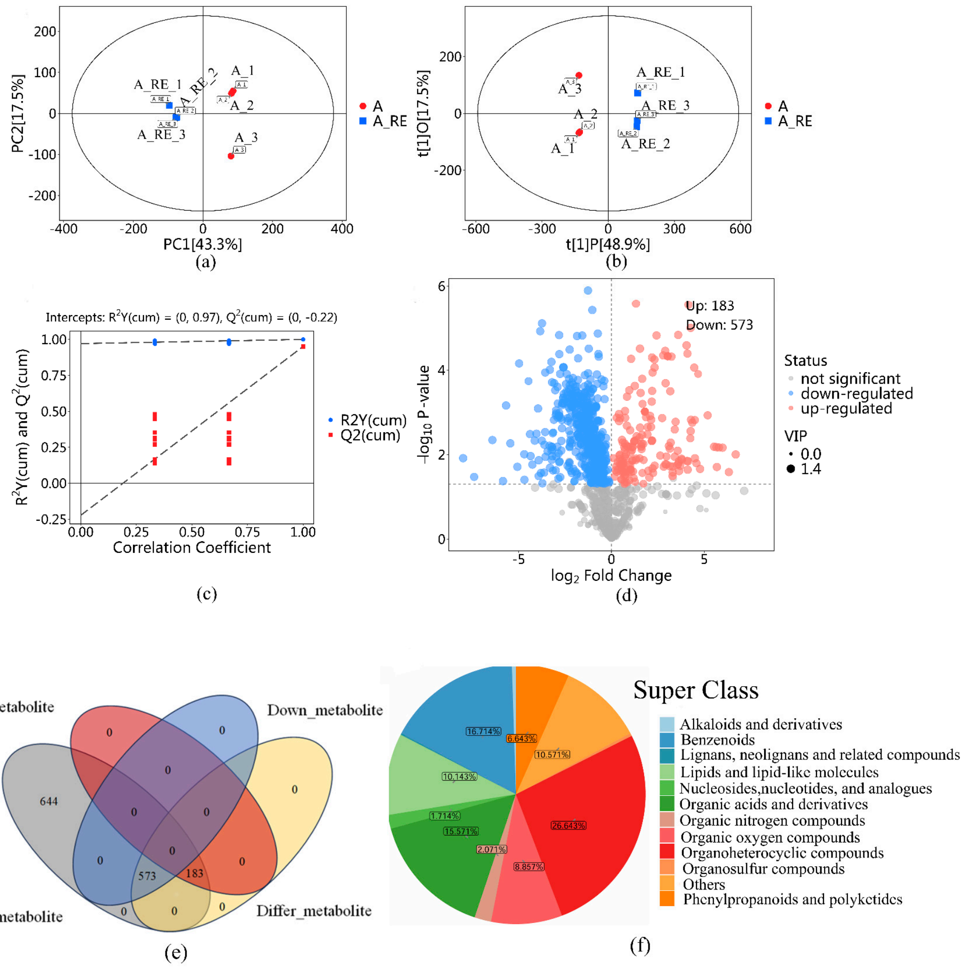

a: Principal component plot (PCA);b: partial least square discriminant analysis plot (PLS-DA);c: PLS-DA permutation test diagram; d: volcano plot; e: Venn diagram; f: statistics of different types of metabolites.

**Figure S6** Metabolomic statistical analysis of *A. niger*.

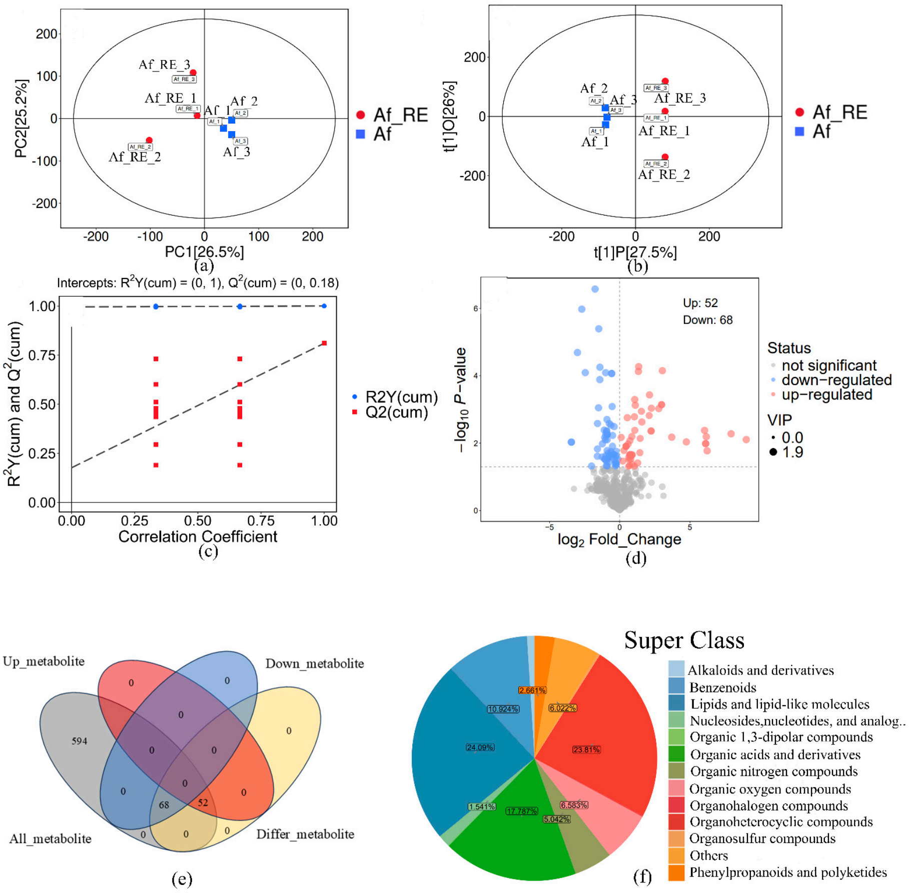


a: Principal component plot (PCA); b: partial least square discriminant analysis plot (PLS-DA); c: PLS-DA permutation test diagram; d: volcano plot; e: Venn diagram; f: statistics of different types of metabolites.

**Figure S7** Metabolomic statistical analysis of *A. ferrooxidans*.

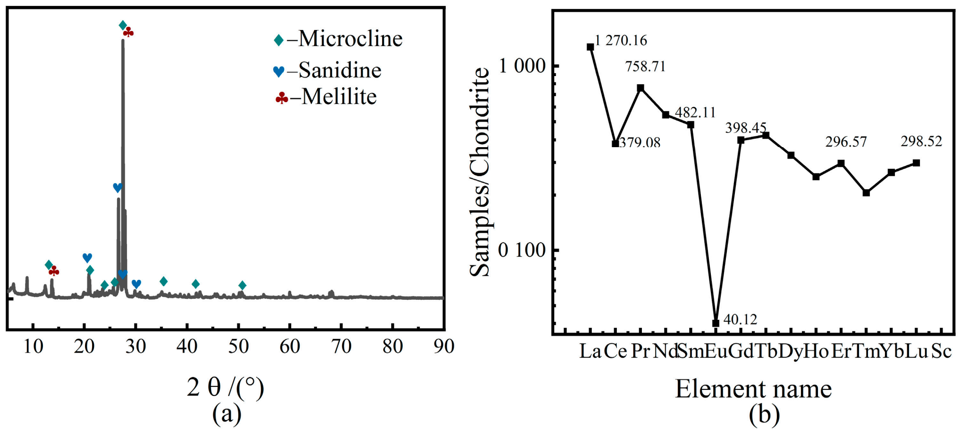


**Figure S8** XRD pattern (a) and rare earth element profiles (b) of primary rare earth ores.

Table S1 PLS model parameters.

| Type | A | N | R2X(cum) | R2Y(cum) | Q2(cum) | Title |
|---|---|---|---|---|---|---|
| OPLS-DA | 1+1+0 | 6 | 0.664 | 1 | 0.951 | A_REvsA |
| OPLS-DA | 1+1+0 | 6 | 0.534 | 1 | 0.81 | Af_REvsAf |

Table S2 Organic acids and derivatives of main 50 differentially abundant metabolites.

| *A. niger* | | *A. ferrooxidans* | |
|---|---|---|---|
| Organic acids and derivatives | | Organic acids and derivatives | |
| Proline | down | 4-Aminobutyric acid (GABA) | up |
| Betaine | down | Glutamate | down |
| N-Acetylhistamine | down | Succinate | up |
| 4-Aminobutyric acid (GABA) | down | alpha-Ketoglutaric acid (alpha-KG) | down |
| Saccharopine | down | Lactate | down |
| Glutamate | down | 2-Hydroxyethanesulfonic acid | down |
| trans-Aconitic acid | down | 4-Oxohexanoic acid | up |
| Malic acid | down | Methylmalonic acid | up |
| 4-Guanidinobutyric acid | up | | |
| N-Acetylputrescine | up | | |
| 2-Hydroxyglutaric acid | down | | |
| Taurine | up | | |
| Ethyl acetoacetate | down | | |
| cis-Aconitic acid | down | | |
| alpha-Ketoglutaric acid (alpha-KG) | down | | |

Table S3 Organic heterocyclic compounds of main 50 differentially abundant metabolites.

| *A. niger* | | *A. ferrooxidans* | |
|---|---|---|---|
| Organic heterocyclic compounds | | Organic heterocyclic compounds | |
| Imidazoleacetic acid | down | 6-(Dimethylamino)purine | up |
| FAPy-adenine | down | N-(3-Methylbut-2-EN-1-YL)- | up |
| | | 9H-purin-6-amine | down |
| 2-Methylthiazolidine | up | 2-Piperidone | down |
| Riboflavin | down | 4-Methyl-5-thiazoleethanol | down |
| Adenine | down | Nicotinate | up |
| 2-Aminopurine | down | 1-Methylguanine | up |
| 2-Piperidone | down | Adenine | up |
| 5-Methylcytosine | up | 9-Ethyladenine | down |
| | | Isonicotinic acid | up |
| | | Guanine | up |
| | | Xanthine | up |
| | | Diazoxide | up |
| | | 2-Aminopurine | up |
| | | 3-Methyladenine | up |
| | | Zoxazolamine | down |
| | | 2-Hydroxyadenine | up |
| | | Oxypurinol | up |
| | | 9-Methyluric acid | up |

Table S4 Lipids and lipid-like molecules of main 50 differentially abundant metabolites.

| *A. niger* | | *A. ferrooxidans* | |
|---|---|---|---|
| Lipids and lipid-like molecules | | Lipids and lipid-like molecules | |
| Glycerophosphocholine | down | Caplamin | down |
| Butanoic acid | down | Isocaproic acid | up |
| Isovaleric acid | down | Caproic acid | up |
| Valeric acid | down | Glycerophosphoethanolamine | down |
| 3-Hydroxybutyrylcarnitine (Car(4:0-O)) | down | 5-Hydroxyvalproic acid | down |
| Acetylcarnitine (Car(2:0)) | down | 5-ketocaproate | up |
| 3-Hydroxyisovaleric acid | down | Ethylmalonic acid | down |
| | | 2-Hydroxyoctanoic acid | down |
| | | PC(18:1(9Z)/18:1(9Z)) | down |
| | | PC(18:1(6Z)/18:1(6Z)) | down |
| | | PC(18:1(9E)/18:1(9E) | down |

Table S5 Benzenoids of main 50 differentially abundant metabolites.

| *A. niger* Benzenoids | | *A. ferrooxidans* Benzenoids | |
|---|---|---|---|
| 4-Hydroxyphenylacetic acid | down | 3-Hydroxybenzoic acid | down |
| 4-Hydroxyphenethylalcohol | down | 4-Hydroxybenzoic acid | down |
| Propyl paraben | up | | |
| 4-Ethylresorcinol | down | | |
| Terephthalic-Acid | up | | |
| 3-Hydroxybenzoic acid | down | | |
| Salicylamide | down | | |
| Ethyl 4-hydroxybenzoate | up | | |
| Ethyl 3-hydroxybenzoate | up | | |
| 4-Hydroxybenzoic acid | down | | |

Table S6 Nucleosides, nucleotides, and analogs of main 50 differentially abundant metabolites.

| *A. niger* Nucleosides, nucleotides, and analogues | | *A. ferrooxidans* Nucleosides, nucleotides, and analogues | |
|---|---|---|---|
| Cytidine | up | Pseudouridine | down |
| Adenosine | up | 5'-Methylthioadenosine | down |
| 2'-O-Methyladenosine | down | | |
| 3'-O-Methyladenosine | down | | |
| Adenosine 3',5'-cyclic phosphate (cAMP) | up | | |

Table S7 Main components of ionic rare earth minerals (analyzed in oxides)

| Composition | Mass fraction/% | Composition | Mass fraction/% |
|---|---|---|---|
| $SiO_2$ | 60.939 | $MnO_2$ | 0.081 |
| $Al_2O_3$ | 27.324 | $Rb_2O$ | 0.054 |
| $K_2O$ | 6.472 | $ZrO_2$ | 0.037 |
| $Fe_2O_3$ | 3.152 | $P_2O_5$ | 0.036 |
| $Na_2O$ | 0.931 | $Cr_2O_3$ | 0.031 |
| MgO | 0.399 | $SO_3$ | 0.028 |
| $TiO_2$ | 0.30 | PbO | 0.015 |
| CaO | 0.19 | ZnO | 0.012 |

Table S8 PDA, fermentation medium and 9K medium.

| PDA medium Ingredient | Content | fermentation medium | Content | 9K medium Ingredient | Content |
|---|---|---|---|---|---|
| glucose | 20.00 g | Glucose | 100.00 | $MgSO_4 \cdot 7H_2$ | 0.50 g |
| $KH_2PO_4$ | 3.00 g | Yeast | 1.00 g | $K_2HPO_4$ | 0.50 g |
| $MgSO_4 \cdot H_2$ | 1.50 g | $(NH_4)_2SO_4$ | 1.00 g | KCl | 0.10 g |
| thiamine | 8.00 mg | $NaNO_3$ | 1.00 g | $(NH_4)_2SO_4$ | 3.00 g |
| AGAR | 15.00 g | $KH_2PO_4$ | 1.00 g | $Ca(NO_3)_2$ | 0.01 g |
| 20% potato | 1000 mL | $MgSO_4 \cdot 7H_2$ | 0.50 g | Deionized | 1000 mL |
| | | $Fe_2(SO_4)_3$ | 0.007 g | | |
| | | Deionized | 1000 | | |
